# Delayed Diagnosis of Blunt Ureteral Injury following Motor Vehicle Collision

**DOI:** 10.1155/2023/8869634

**Published:** 2023-05-05

**Authors:** Alexander Canales, Harsh Desai

**Affiliations:** Allegheny Health Network, Forbes Regional Hospital, 2570 Haymaker Road, Monroeville, PA 15146, USA

## Abstract

**Background:**

A 19-year-old male requiring emergency surgery after presenting to the emergency department (ED) as a trauma activation status post-motor vehicle collision. *Summary.* The patient presented to the ED after a motor vehicle collision. He was taken emergently to the operating room after finding hemoperitoneum on computerized tomography scan without evidence of solid organ injury. Significant small and large bowel injuries were discovered requiring resection and anastomosis. The patient had an uneventful post-operative recovery and was discharged home. He was later re-admitted to the hospital with a large pelvic abscess and a left mid-ureteral stricture causing hydronephrosis. The abscess was treated with antibiotics, and the left ureteral injury was treated with a nephrostomy tube and stent placement. He made a full recovery after hospital re-admission and a delay in diagnosis of blunt ureteral injury.

**Conclusion:**

Patients involved in motor vehicle collisions are at risk of multi-system trauma including genito-urinary injuries. A small percentage of these patients may present with blunt ureteral injuries. A high index of suspicion is required to make an early diagnosis. Earlier diagnosis may help to prevent morbidity.

## 1. Case Description

Ureteral injury is an exceedingly rare entity accounting for less than 4% of penetrating injuries and less than 1% of blunt trauma [1]. Injuries usually result from deceleration mechanisms and often have associated organ injuries [2, 3]. These injuries can be subtle as demonstrated by this case of a mid-ureter injury with a delayed presentation.

**Figure 1:**
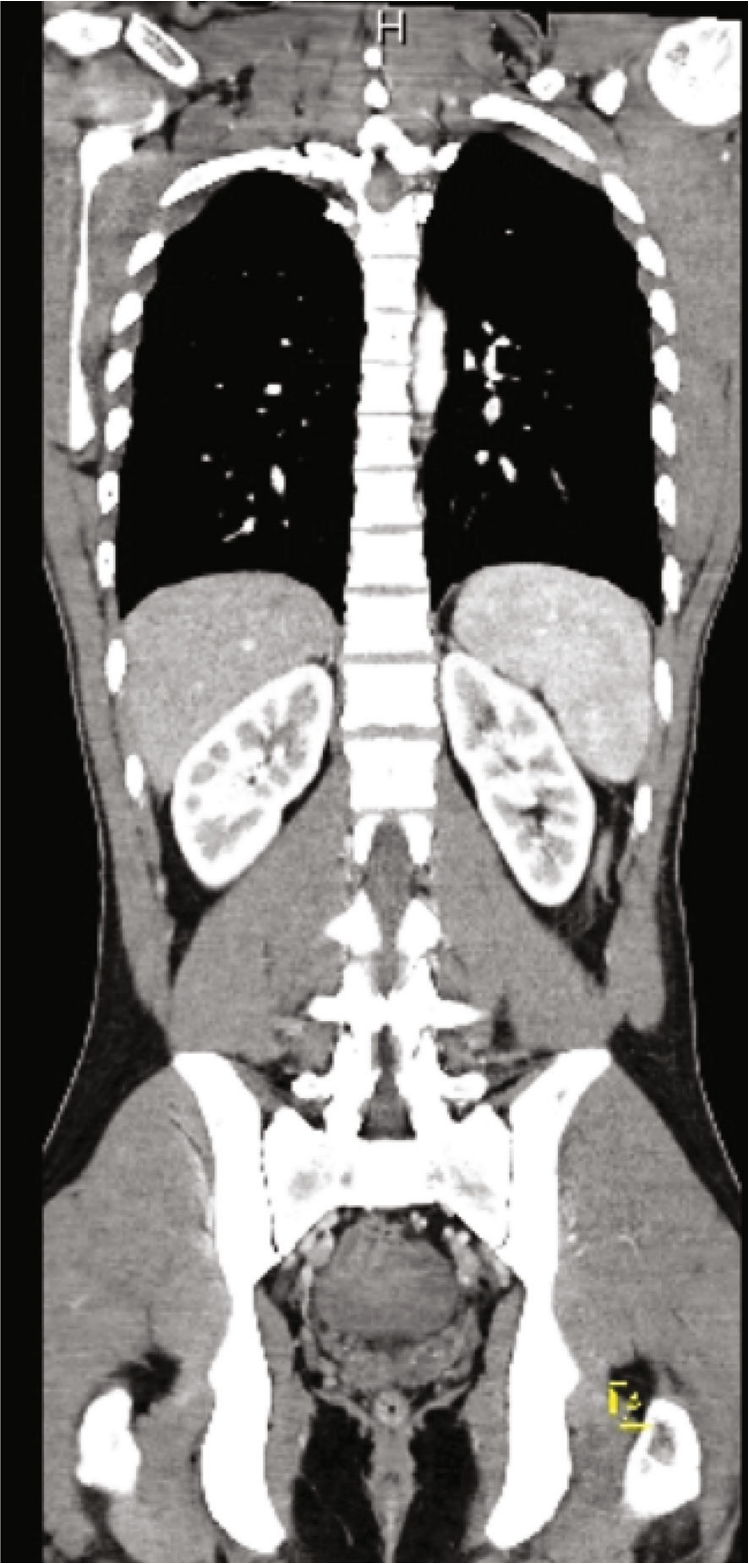
Coronal view from initial trauma pan-scan showing atraumatic kidneys, and hemoperitoneum is noted. Ureters not visualized due to lack of delayed phase.

This case involves a 19-year-old male who presented status post head-on motor vehicle collision. He self-extricated and started complaining of left-sided abdominal pain and left knee pain on scene. He was transported to the trauma center for evaluation. On primary survey, he did not have any airway or breathing issues. He was noted to be Glasgow Coma Scale 15 and hemodynamically stable. On secondary survey, he had a positive seat belt sign with significant abdominal tenderness to palpation and a 3 cm left knee laceration. He was taken to computerized tomography (CT) for a trauma pan-scan, which included a CT brain, cervical/thoracic/lumbar spine, and a CT angiogram of the neck, chest, abdomen, and pelvis. CT showed hemoperitoneum without evidence of solid organ injury (Figure 1). A delayed phase was not completed at this time, because it was not specifically requested. Therefore, the ureters were not visualized. The only other injury noted on imaging was a right first metacarpal fracture. Due to the presence of a seat belt sign, hemoperitoneum without evidence of solid organ injury and ongoing severe abdominal pain, the decision was made to take the patient to the operating room for an exploratory laparotomy. Pertinent findings included a 10 cm de-vascularized segment of ileum and a de-vascularized segment of the sigmoid colon. Both sections of bowel were resected. During the case, the left ureter was identified just proximal to the left common iliac artery prior to resection of the sigmoid colon. The ureter was not skeletonized. Since the patient was otherwise healthy, young, and hemodynamically stable, both segments of bowel were re-anastomosed. The small bowel was anastomosed in a side-to-side fashion, functional end-to-end type fashion, and the colon was anastomosed in a side-to-side isoperistaltic fashion. The abdomen was closed, and the patient was admitted to the floor. His initial post-operative course was completely unremarkable, including all bloodwork. He neither had gross nor microhematuria, including pre-operatively. His creatinine was within normal limits. He was discharged home on post-operative day 4.

**Figure 2:**
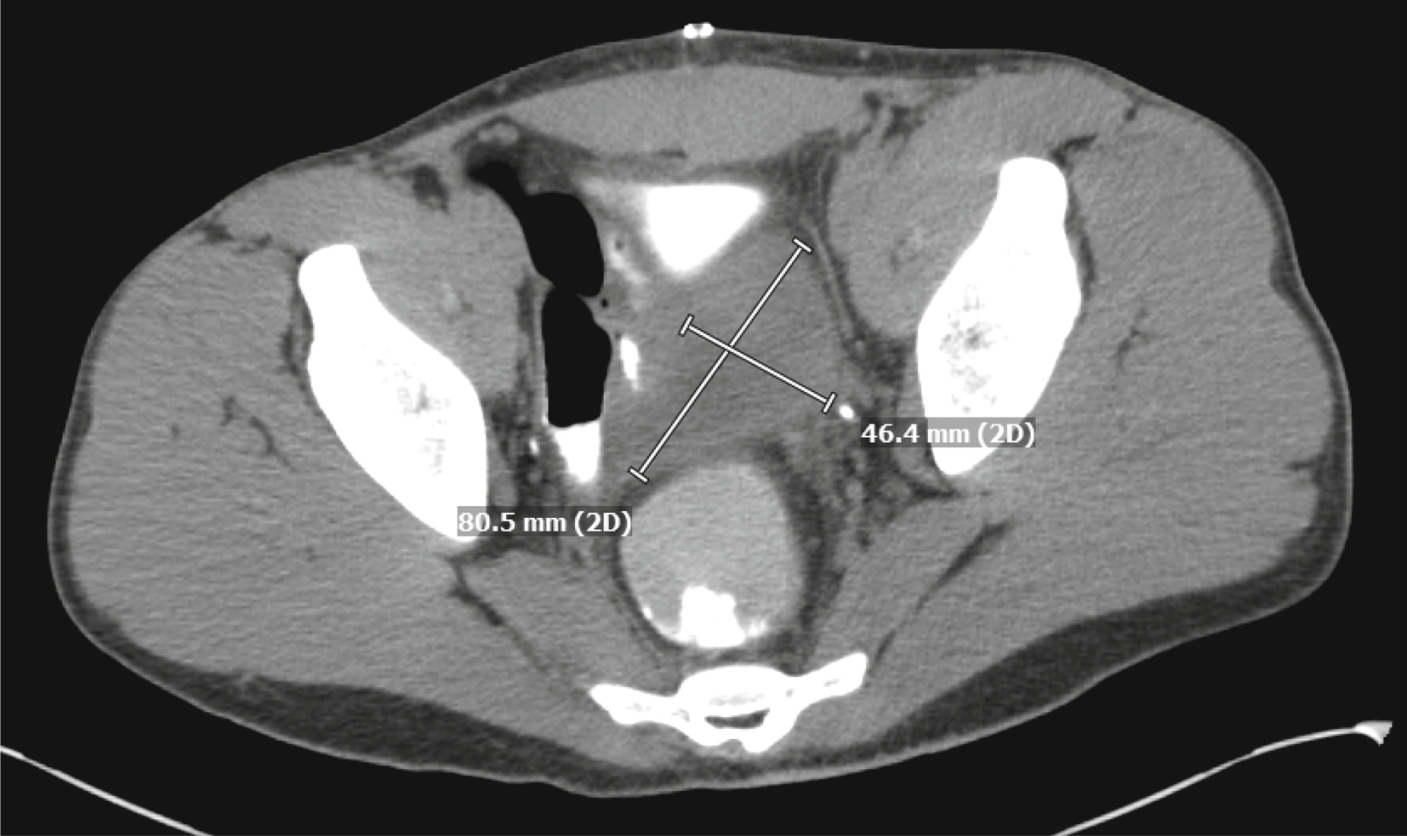
Axial view of the recto-vesicular pouch abscess measuring approximately 8 cm × 4.6 cm, at its largest diameter.

**Figure 3:**
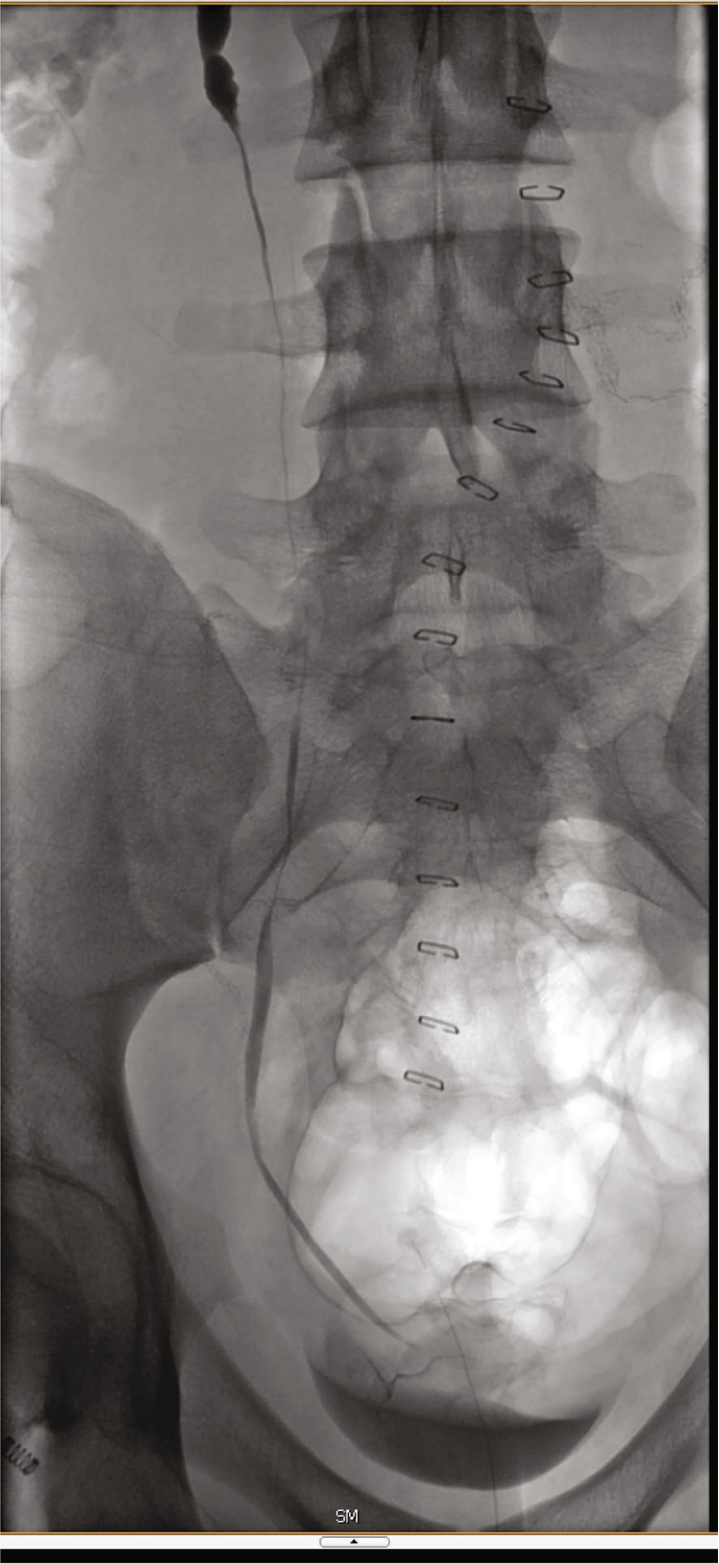
Posteroanterior view of left nephrogram showing an 8 cm mid-ureter stricture and no evidence of contrast extravasation.

On post-operative day 10, the patient returned to the emergency department with abdominal pain. He was afebrile, with a leukocytosis of 13 k/mcL and increased creatinine at 1.24 mg/dL. Urinalysis was completely negative including lack of hematuria. On CT of the abdomen with intravenous (IV) and oral contrast, he was found to have an 8 cm × 4.6 cm abscess in the recto-vesicular pouch (Figure 2). Immediate follow-up CT with rectal contrast was negative for contrast extravasation. However, this scan also served as a delayed phase demonstrating evidence of mild left hydronephrosis and hydroureter. The patient was admitted to the floor and started on antibiotics. Because of the abscess and a concern for ureteral injury, Interventional Radiology (IR) and Urology were consulted. There was concern for ureteral injury because of the elevated creatinine, history of prior surgery with known risks factors for ureteral injury, and the presence of hydronephrosis/hydroureter. The plan per urology was to drain the abscess and then attempt a retrograde pyelogram and stent placement. Their concern was that the abscess was large enough to extrinsically compress the ureter and make a retrograde approach challenging. IR attempted drainage of the abscess but despite multiple different approaches, were unable to find a safe window. Therefore, cultures and a creatinine level were not able to be obtained from the abscess. This also meant that the possible extrinsic compression of the ureter was not remedied. A left pyelogram was then performed by IR that showed an 8 cm mid-ureteral stricture and no extravasation (Figure 3). A nephrostomy tube was placed during this procedure. A few days later, a left ureteral stent was placed. The rest of his re-admission was unremarkable. He was discharged home on post-operative day 17.

A follow-up renal ultrasound 3 weeks after nephrostomy and stent placement showed resolution of the hydronephrosis. An antegrade pyelogram was performed at 4 weeks, which confirmed resolution of the hydronephrosis. The nephrostomy tube was removed at this time. At 6 weeks, a retrograde pyelogram was performed with stent removal. Complete resolution of the ureteral stricture and resultant hydronephrosis was documented. Imaging is not available from these procedures. He continues to recover without further complications.

## 2. Discussion

Due to their rarity and sometimes subtle signs and symptoms, blunt ureteral injuries can present in a delayed fashion [4]. It is important to recognize these injuries early, as delayed diagnosis can result in significant morbidities, such as urinoma, abscess, ureteral stricture, potential loss of kidney function, and even death [1, 5, 6]. A high index of suspicion is required with certain trauma mechanisms, such as accidents with hyperextension or deceleration mechanisms [5].

In addition to the mechanism of injury, certain signs or symptoms can suggest a ureteral injury. The presence of hematuria can be considered a sign of ureteral trauma. However, the lack of hematuria is an unreliable sign to exclude injury [5, 6]. Additional signs of injury can be flank pain or ecchymosis [6]. Diagnosis of ureteral injuries can be made with various imaging modalities and direct inspection of the ureter during surgical exploration. CT scan with a 5-minute delayed phase, antegrade, and retrograde pyelograms are recognized as accurate means of diagnosis [1, 3, 5, 6]. A retrograde pyelogram is considered the most accurate [1, 3, 5–7]. When there are other findings on CT with IV contrast, such as mild perinephric stranding, low density retroperitoneal fluid around the genito-urinary tract, and perinephric hematomas, and a delayed phase should be ordered [8].

Ureteral injuries are graded according to the American Association for Surgery of Trauma (AAST) Organ Injury Scale. A grade 1 injury is a contusion or hematoma without devascularization. A grade 2 injury is a <50% circumference laceration. A grade 3 injury is a >50% circumference laceration. A grade 4 injury is a complete transection of the ureter with <2 cm devascularization. A grade 5 injury is an avulsion of the ureter with >2 cm devascularization. Grades 1–3 are advanced by 1 for bilateral injuries [9].

The most common injury pattern seen in motor vehicle collisions is disruption of the ureteropelvic junction [5]. Although, this is usually seen in children and is classified as a kidney injury according to the AAST Organ Injury Scale [5, 11].

Treatment options correlate with the AAST grade, site of injury, associated injuries, and whether the injury is diagnosed in the acute or delayed setting [6]. When identified, a multi-disciplinary approach to management, may produce better outcomes [10]. The primary objective of treatment is to preserve renal function. Treatment includes stenting, diversion, or surgical repair. If surgical repair is performed, the repair should be tension free and use healthy tissue for anastomosis [6]. Delayed diagnosis is best managed by urinary diversion and/or stent placement. This is followed by a reconstructive procedure, if necessary [5]. Follow-up is largely provider dependent and depends on the extent of injury and treatment provided. A Foley should be left in place for 10 days if the bladder is involved in the repair. Stents are usually left for 6 weeks [1]. Prognosis of treatment largely depends on the timing of diagnosis. Earlier identified injuries seem to correlate with better outcomes than those with delayed diagnoses [1].

This case serves as an example of how a patient involved in a motor vehicle collision is at risk of blunt ureteral injury. The patient had many risk factors for ureteral stricture including the trauma itself, surgery with mobilization of the left colon, and a large post-operative abscess. However, during the surgery only gentle traction was used, and the ureter was not skeletonized. The abscess was present at the lower portion of the stricture but did not explain the length of segment involved. It is the authors' opinion that this stricture was a result of a contusion to the ureter caused by the motor vehicle collision. A high index of suspicion at admission may have made the diagnosis earlier. It is possible that earlier diagnosis may have prevented ureteral stricture and the need for urinary diversion. Despite ultimately having a favorable outcome, delayed diagnosis still resulted in hospital re-admission and additional procedures.

## 3. Conclusion

Patients involved in motor vehicle collisions are at risk of multi-system trauma including genito-urinary injuries. A small percentage of these patients may present with blunt ureteral injuries. A high index of suspicion is required to make an early diagnosis. Earlier diagnosis may help to prevent morbidity.

## Data Availability

Epic EHR was used for the case description.
